# High aspect ZnO nanorod growth over electrodeposited tubes for photocatalytic degradation of EtBr dye

**DOI:** 10.1039/d0ra08124h

**Published:** 2021-01-05

**Authors:** Hrudaya Jyoti Biswal, Anshul Yadav, Pandu R. Vundavilli, Ankur Gupta

**Affiliations:** School of Mechanical Sciences, Indian Institute of Technology Bhubaneswar Odisha 752050 India ankurgupta@iitj.ac.in; Membrane Science and Separation Technology Division, CSIR-Central Salt and Marine Chemicals Research Institute Bhavnagar 364002 Gujarat India; Department of Mechanical Engineering, Indian Institute of Technology Jodhpur Rajasthan 342037 India

## Abstract

In this work, vertically grown rod type ZnO nanostructures have been synthesized on metallic nickel tube films fabricated through the cost-effective process of electroforming. The use of tubular metal substrates for the growth of ZnO nanorods has been found to be advantageous for the photocatalytic degradation of EtBr dye because of their high surface area-to-volume ratio. The nickel tubes with ZnO nanorods were characterized using X-ray diffraction (XRD) and field emission scanning electron microscopy (FESEM). The developed system was utilized for the photocatalytic degradation of EtBr dye and its efficacy was revealed through the comprehensive mineralization of the dye within 150 min. The mechanism of the degradation process has been revealed through total organic carbon (TOC), chemical oxygen demand (COD) and high-performance liquid chromatography (HPLC) studies. A substantially lower amount of the photocatalyst has been used because of the homogeneously distributed growth of nanorods on the substrate. Density functional theory-based analysis has also been performed to study the photocatalytic degradation and adsorption properties of EtBr on ZnO nanorods. Using first principles DFT theory, geometry optimizations and vibrational analysis are performed which show a negative charge transfer from the substrate to the photocatalyst. For the first time this article reports the use of DFT analysis for investigating the adsorption of EtBr on ZnO nanorods, and the experimental growth of nanorods over electroformed Ni tubes.

## Abstract 2

In this work, vertically grown rod type ZnO nanostructures have been synthesized on metallic nickel tube films fabricated through the cost-effective process of electroforming..

## Graphical abstract

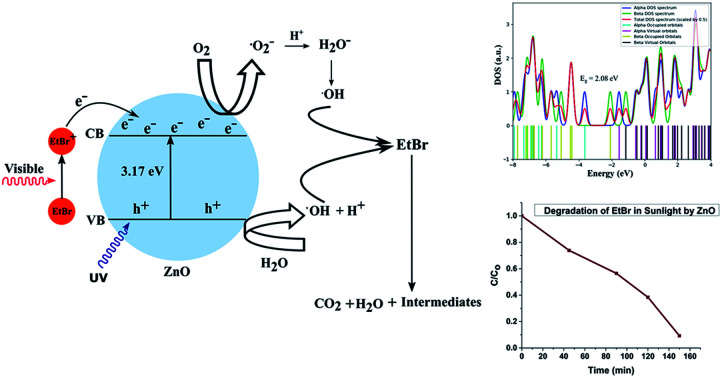

## 1 Introduction

**Fig. 1:**
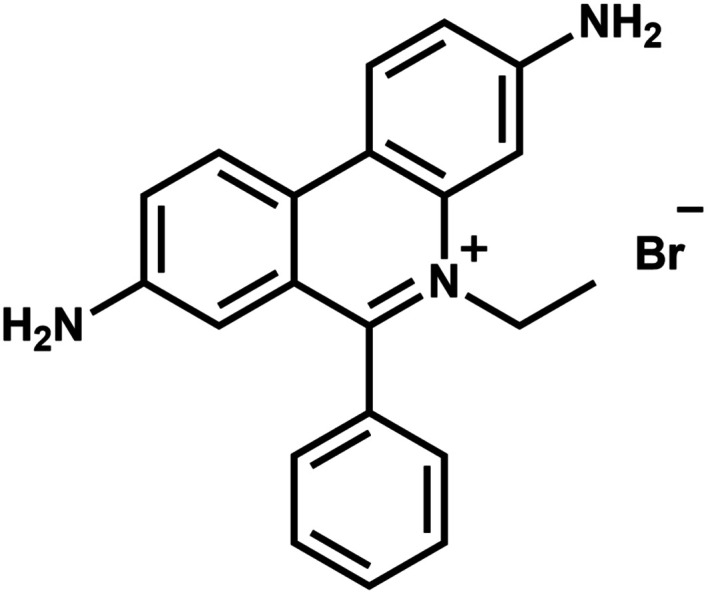
Ethidium bromide.

Ethidium bromide (EtBr, 3,8-diamino-6-phenyl-5-ethylphenanthridine bromide, as shown in Fig. 1) is an aromatic, non-volatile compound having a tricyclic structure with an aniline on either side of a pyridine. The resemblance of the EtBr ring to the ring of DNA bases makes it an efficient non-radioactive marker for visualizing nucleic acids in electrophoretic and other gel-based techniques. The formation of van der Waals contacts with the base pairs of DNA and RNA is responsible for the mutagenic property of EtBr. The widespread popularity of the same chemical as a staining agent for nucleic acids makes its disposal from chemical laboratories a serious concern. Various EtBr elimination processes like chemical degradation, extraction, adsorption and bleaching^1^*etc.* have been adopted. But it was not possible to achieve complete degradation using these processes. Also, disposal techniques like bleaching give out more toxic by-products than EtBr itself. Photocatalytic degradation has emerged as an economical and green technology utilizing the renewable source of energy with advantages like high oxidation efficiency,^2^ complete degradation capability and low levels of toxic by-products. There have been many studies in recent years which show semiconductor oxides as effective photocatalytic agents for degrading organic pollutants. Various metal oxide semiconductors *viz.*, TiO_2_, SnO_2_ and ZnO *etc.* have been studied and found to be suitable for photocatalytic degradation due to their large band gap and their ease of fabrication into different shapes.^3–6^ Among the semiconductors, ZnO has been used extensively due to its non-toxicity, good photocatalytic efficiency, better sensing ability^43^ and low cost. A wide band gap (3.37 eV) and large exciton binding energy (60 mV) makes it one of the promising materials for photocatalysis. Many previous studies have shown ZnO has higher photocatalytic efficiency than TiO_2_ under certain conditions^7–9^ because of its high absorption capacity over a wide UV range. The utilization of ZnO nanostructures for photodegradation has also taken centre stage in recent times.^10^ The photocatalytic degradation of EtBr has been investigated by various research groups using TiO_2_ and metal doped TiO_2_ as catalysts.^11–13^ Among the numerous degradation methods, no work has been found to focus on the photocatalytic degradation of EtBr using ZnO as the catalyst.

The degradation of pollutants in the presence of semiconducting oxides depends largely on the morphology of the catalyst. An enhanced decomposition rate requires a high surface-to-volume ratio as the photocatalytic reaction takes place at the surface of the catalyst. The photocatalysts work by generating reactive hydroxyl radicals which oxidize the toxic pollutants and decompose them into smaller fragments. The production of the hydroxyl radicals is prompted by the creation of electron–hole pairs due to the irradiation induced photoexcitation of ZnO. A factor influencing this photocatalysis is the recombination of photo induced electron–hole pairs which adversely affects the photocatalytic yield. But when the size of the catalyst approaches the nanoscale, fast arrival at the reaction sites promotes a decrease in the recombination of electron–hole pairs. In some previous works, ZnO 1D nanostructures have been shown to exhibit enhanced photoactivity.^39–42^ Another difficulty after the photocatalytic reaction is the removal of suspended nano-sized catalysts from the water medium.^14,15^ The recyclability of the catalyst for repeated usage is directly dependent on the recovery of ZnO from the water medium. Some researchers have tried to solve this issue by tagging magnetic particles with semiconductor oxide nanoparticles.^16^ Hence the template-based growth and development of ZnO nanostructures has taken centre stage for the dye degradation process. Template or substrate-based growth renders a larger surface area for the catalytic reaction as well as enhancing the dispersion of the metal oxide semiconductor nanostructures. The advantage that substrate-based nanostructures have over nanoparticles in powdered form is that these prevent the agglomeration of particles thus the interface area between the catalysts and the reaction solution is maintained. As the turbidity of the particles increases, the penetration capacity of the light energy decreases thus reducing the photocatalytic activity. Metal-based substrates cater to the possibilities of being used both for low-temperature as well as high-temperature growth techniques. Besides, if metals which have magnetic properties are used as substrates they are easier to retrieve from the solution and reuse. Finally, there has been no cited work reporting ZnO nanorod growth on a metal substrate, except that of Zainelabdin *et al.*^17^ In this paper, we report the synthesis and characterization of ZnO nanostructures on nickel micro-tubes. The micro-tubes not only act as a substrate but also provide a higher surface area-to-volume ratio for the development of nanorods. The tubular structure takes care of the space constraint for the reaction too. The interface between the metal and the semiconductor is also expected to separate the charge and enhance the photocatalytic activity.

A quantum cluster technique for the adsorption of organic molecules/ions may be used for simulation and analysis using density functional theory (DFT) calculations. DFT calculations for organic molecule adsorption on photocatalyst surfaces and some DFT studies on ZnO nanostructures have already been carried out^18–20^ and different properties have been calculated such as adsorption energies and geometries. However, DFT methods for researching the adsorption of EtBr on ZnO nanorods have not been used till now. In this work, we have carried out the study of the photocatalytic degradation of EtBr on ZnO nanorods, theoretically through first principles DFT calculations.

## 2 Materials and methods

In order to fabricate the metallic tubular films and for the subsequent development of nanoseeds and nanorods, the chemical reagents used are nickel sulphate (NiSO_4_·6H_2_O), nickel chloride (NiCl_2_·6H_2_O), boric acid (H_3_BO_3_), zinc acetate dihydrate (Zn(CH_3_COO)_2_·2H_2_O), zinc nitrate hexahydrate (Zn(NO_3_)_2_·6H_2_O), hexamethylenetetramine (HMTA, (CH_2_)_6_N_4_), potassium hydroxide (KOH) and acetone. All these chemicals were procured from Central Drug House (CDH), Delhi, India while isopropyl alcohol (IPA) and glacial acetic acid (>99.99%) were purchased from Merck Limited, Mumbai. Other chemicals such as acetonitrile (HPLC grade) and HPLC water were procured from Avra Synthesis, Hyderabad, India. The chemicals were used without further purification and deionized water was utilized to prepare all of the aqueous solutions. The chemicals were used as per the details given in the following sections. Ultrasonication and mechanical stirring were used for dispersion and solution preparation. A hot plate with a temperature control unit and a magnetic stirrer (IKA RCT Basic model, range: 0/50–1700 rpm, 50–380 °C) was employed in this work. A pulse generator (Scientific SM5035, range: 20 mHz–20 MHz) was used in the electroforming process of tube fabrication. The following detailed steps were adhered to for the preparation of the metallic film and ZnO nanorods.

### 2.1 Fabrication of tube substrates with electroforming setup

**Fig. 2:**
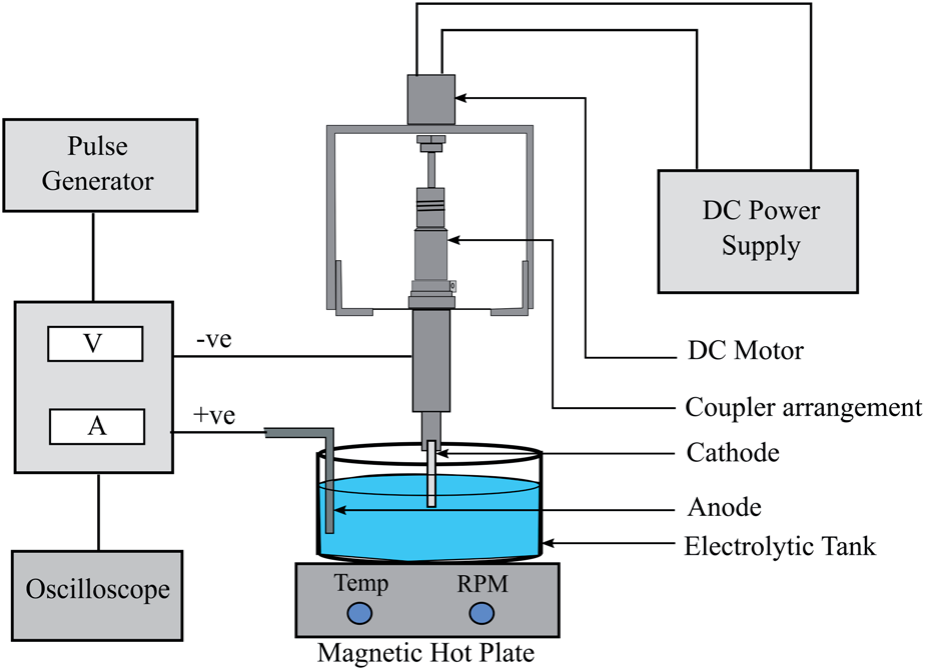
Schematic diagram of the electroforming system.

In electroforming, the material build-up takes place atom-by-atom on a pre-shaped cathode which is a negative replica of the part to be produced. The fabrication of the metallic (nickel) tubular film substrates was carried out through the process of electroforming. The electroforming system for microtube fabrication was designed and developed by retrofitting the table top drilling equipment. The schematic diagram of the experimental equipment is shown in Fig. 2. Pulse current waveform was utilized instead of direct current for better control of the thickness and properties of the films.^44,45^ A constant potential was applied during the on-time that contributed towards mass deposition while the off-time helped the removal of excess metal ions. Ni wire was used as the anode and aluminum mandrels with a 2.9 mm diameter were used as the cathode. The aluminium mandrels were treated in acetone and then washed in distilled water. The mandrel material used for the electroforming process should be selected based on the nature of the etchant: the etchant should be able to dissolve the mandrel material without affecting the electroformed part. Mostly copper is the preferable choice as the mandrel material due to its higher conductivity and commercial availability in various manufactured wires and tubes. But in the case of nickel as an electroform, aluminium could be the preferred mandrel material, due to its better selective etching capability than copper in the presence of nickel.^21^ The deposition process was carried out in the electrolyte cell of a 600 mL Watts bath having a composition of 300 g L^−1^ of NiSO_4_·6H_2_O, 30 g L^−1^ of NiCl_2_·6H_2_O and 35 g L^−1^ of H_3_BO_3_. Besides acting as a secondary source of nickel ions, nickel chloride also contributes towards enhancing the conductivity of the solution. The pH value and the temperature of the bath were maintained at 4.5 and 54 °C, respectively. The hot plate was used to control the temperature as well as to agitate the bath. The desired microtube length was fixed by placing insulating tapes on both sides of the mandrel. The set of experiments carried out earlier was utilized to optimize and choose the parameters (duty cycle and time of deposition) suitable for the present work. After the deposition of Ni upon the mandrel, the aluminium was chemically etched in KOH for the removal of the electroformed part. A 2 M solution of KOH was prepared by stirring it with a magnetic stirrer whilst maintaining a temperature of 40 °C. The surface morphology characterization and crystallite size determination were carried out using an X-ray diffractometer (D8-Advance, Brooker, Germany, Cu-Kα radiation, Bragg–Brenton geometry, 40 kV, 40 mA, 0.015° s^−1^ scan rate). A field emission scanning electron microscope (ZEISS MERLIN compact) in conjunction with EDAX was employed for analysing the coating morphology, tube thickness and elemental analysis. Hardness tests were carried out using Zwick Roell Indentec with a load of 100 gf and a dwell time of 10 s. The average of three readings was recorded for this purpose. A contact-type surface measurement instrument (Mitutoyo SJ-210) was used for the measurement of surface roughness and morphology.

### 2.2 Fabrication of ZnO nanoseeds in and around the tube surface area

**Fig. 3:**
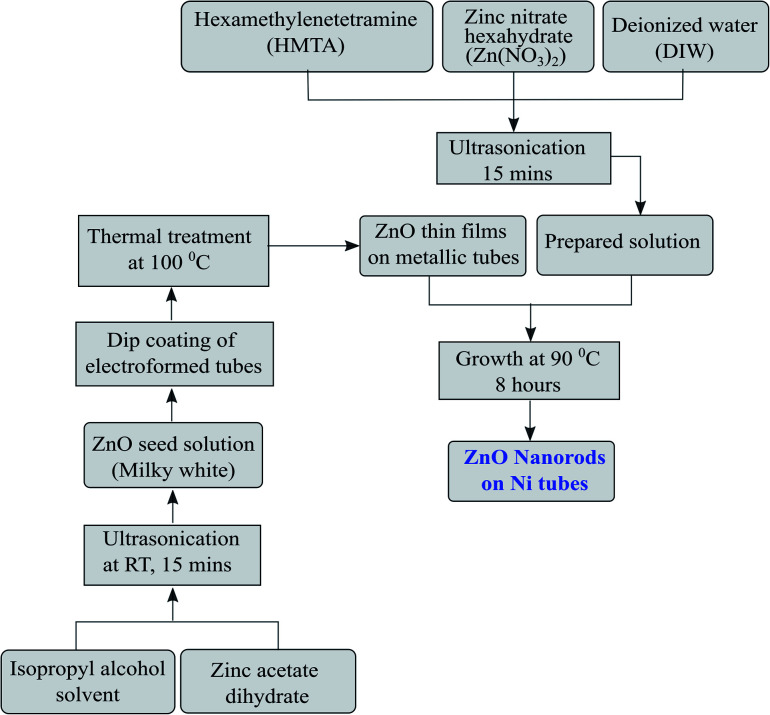
Flow diagram of the ZnO nanorod development and growth process.

The development of ZnO nanorods on the metallic substrate through the process explained below is an eco-friendly and simple technique and results in a large-area growth of nanorods. To grow 1D ZnO nanorods, the first step is the preparation of ZnO nanoseeds on the tubular substrates. The solution of zinc acetate dihydrate (0.1 M) in isopropyl alcohol was prepared by ultrasonication. The ultrasonication was carried out till the solution turned milky white in colour. The nickel microtubes were rinsed properly in distilled water and subsequently with acetone before being dipped in the zinc acetate solution for the initial growth of nanoseeds. The tubes were then baked using a hot plate at a temperature of 100 °C. The process was repeated five to six times to get a uniform layer thickness of nanoseeds for growth. The high density seeded substrates were utilized to facilitate the growth of ZnO nanorods. The growth step was carried out by preparing a solution mixture of zinc nitrate hexahydrate (0.01 M) and hexamethylenetetramine (HMTA, 0.05 M) in distilled water. The solution was subjected to ultrasonication for homogenization. The substrates were kept inside the Zn atmosphere of the solution for 8 hours at a temperature of 90 °C. The step-wise deposition of the seed layer as well as the growth of the nanorods are shown in Fig. 3. The morphology and dimensions of the grown ZnO nanorods were then analyzed using a field emission scanning electron microscope (ZEISS MERLIN compact).

The growth of ZnO nanorods on the metallic substrate proceeds through a number of chemical reactions. The hydrolysis of HMTA in the solution in the presence of heat releases formaldehyde (HCHO) and ammonia (NH_3_). The released ammonia subsequently assists in the formation of hydroxyl ions which prevent the bulk nucleation of Zn^2+^ in solution. The ammonia further decomposes into OH^−^ and NH_4_^+^. The OH^−^ combines with the Zn^2+^ to form the complex compound of ZnOH_4_^2−^ which dehydrates into ZnO nanorods under the reaction conditions. The step by step reaction equations are given below.Zn(NO_3_)_2_·6H_2_O → Zn^2+^ + 2NO_3_ + 6H_2_OC_6_H_12_N_4_ + 6H_2_O → 6HCHO + 4NH_3_C_6_H_12_N_4_ + 4H_2_O → (CH_2_)_6_(NH)_4_ + 4OH^−^NH_3_ + H_2_O → OH^−^ + NH_4_^+^Zn^2+^ + 4OH^−^ + H_2_O → Zn(OH)_4_^2−^Zn(OH)_4_^2−^ → ZnO + H_2_O + 2OH^−^

### 2.3 Photocatalytic study

**Fig. 4:**
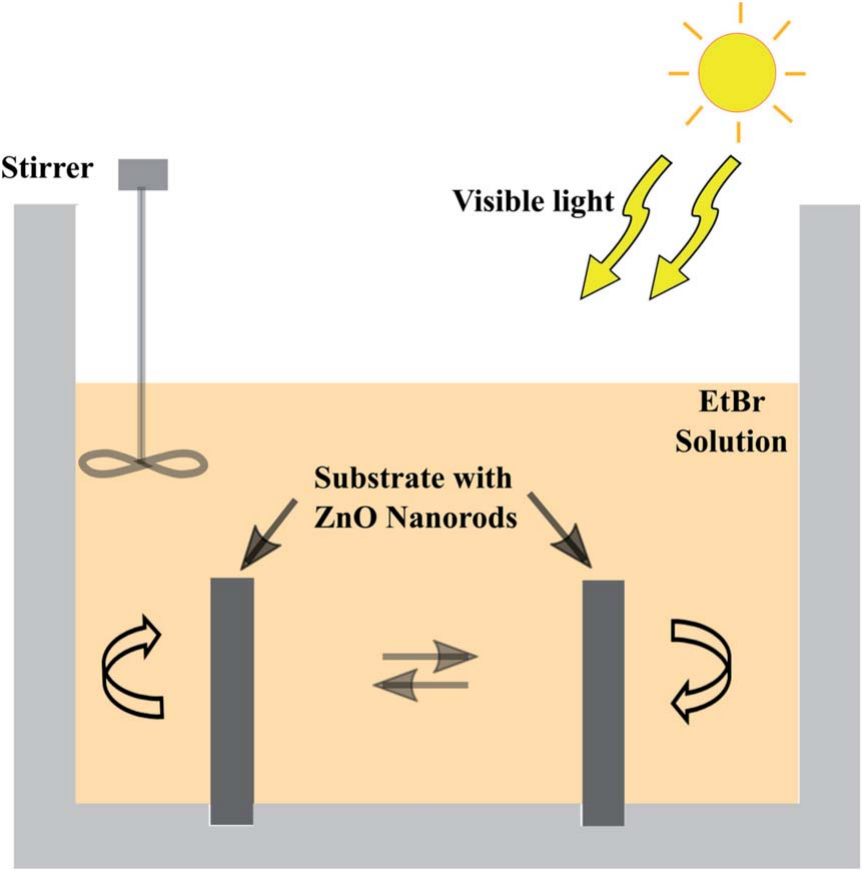
Schematic layout of the photocatalytic degradation setup.

The photocatalytic efficiency of the prepared nanorods on the electroformed tubular films was tested by measuring the rate of degradation of the EtBr (M/S Sigma Aldrich) in aqueous solution using sunlight. An acrylic tank with holes drilled in the base was fabricated in order to hold the tubes upright exposing both the inner and outer surfaces to the organic solution. A 200 mL aqueous solution with a concentration of 10 mg L^−1^ was put in the container and intermittently stirred manually. Two tubular films with a diameter of 2.9 mm and length of 2 cm, densely covered with ZnO nanorods were immersed in the EtBr solution. The experimental setup was kept under sunlight with an average intensity of 144 mW cm^−2^ calculated according to the longitude and latitude. The purpose of this work is to test the efficacy of the grown ZnO nanorods for visible light photocatalysis, thus, solar irradiation was utilized. 3 mL aliquots were taken at regular intervals and their absorbances were measured using a UV-Visible spectrophotometer (Hitachi U-2900) to evaluate the degradation rate of the solution. Fig. 4 depicts the schematic layout of the experimental system with the organic solution.

### 2.4 Analytical methods

The UV-Visible spectra in diffuse reflectance mode was recorded using a Shimadzu UV-2450 (Japan) spectrophotometer for calculating the band gap energy of the grown ZnO nanorods on tubular nickel films. The chemical oxygen demand (COD) measurements for the degraded dye samples at different irradiation times were carried out *via* a closed reflux colorimetric method which subjects the dye samples to potassium dichromate in an acidic solution. For these analyses a COD digester and a UV–Visible spectrophotometer (Hitachi U-2900) were put to use. The total organic carbon (TOC) concentration in the sample was analyzed using a TOC analyzer (TOC-L, Shimadzu) equipped with an automatic sampler (OCT-L, Shimadzu). The degradation process of the EtBr solution was monitored in detail using a high-performance liquid chromatography (HPLC) system (Ultimate 3000, DIONEX, USA) equipped with a pump and a UV detector at 285 nm. A C-18 column (HYPERSIL GOLD 5UM, Thermo scientific) with dimensions 250 mm × 4.6 mm was used for the separation of analytes. The sample was filtered through 0.22 μm filter paper (Thermo Scientific, PVDF). The mobile phase used was acetonitrile and 1% acetic acid (80 : 20 v/v) which was applied at a flow rate of 1 mL (min)^−1^. The injection volume and retention time were 20 μL and 6 minutes respectively. Data acquisition was performed using Chromeleon 7 software.

### 2.5 Computational model

**Fig. 5:**
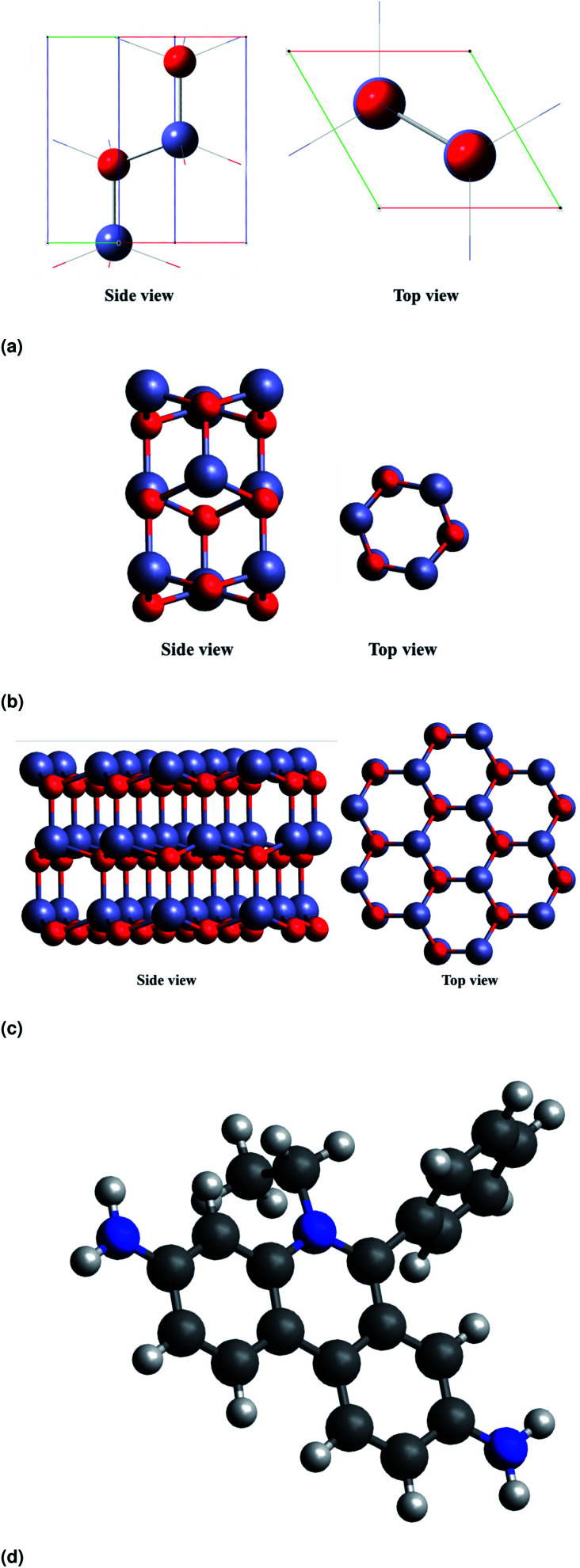
DFT optimised structures of (a) the unit cell of wurtzite ZnO, (b and c) ZnO NRs with different cross-sectional diameters and (d) EtBr^+^ ion.

All calculations reported in the present study were carried out using density functional theory in Quantum ESPRESSO package. The density of states (DOS) plots have been visualized with the help of GaussSum software.^22^ In our study, we have considered ZnO nanorods (NRs) in a hexagonal cross section that are periodic along the [0001] direction. The unit cell of the ZnO nanorod is shown in Fig. 5a.

The unit cell has been optimised using the PBE function^23^ as it is a repeating unit. The HOMO–LUMO energies are common quantum mechanical descriptors,^24^ by which the wide range of chemically-based interactions is regulated. The higher energy difference between the HOMO and LUMO suggests greater kinetic energy and lower chemical reactivity. In analytical frequency calculations with the same level of geometry optimizations, the stationary points are confirmed as minimal (not imaginary frequencies). We have constructed atomistic models of the ZnO nanorods with varied NR diameters. Fig. 5b and c show the side and top view of ZnO NRs with diameters of 3.71 Å, and 9.86 Å respectively (diameters are defined with respect to the position of the oxygen atoms at the surface). Fig. 5d shows the optimized structure of EtBr^+^ using DFT. For simplicity of calculations and not to stress the program, we consider a single ZnO NR unit to study the interaction between ZnO and EtBr. The adsorption energy for EtBr adsorbed on the ZnO NR is computed using the following relationship.*E*_ads_ = *E*(EtBr + ZnO) − [*E*(ZnO) + *E*(EtBr)]where *E*(EtBr + ZnO) represents the electronic energy of the cluster and the adsorbed EtBr, *E*(EtBr) is the electronic energy of the optimized EtBr, and *E*(ZnO) is the electronic energy of the corresponding ZnO NRs.

## 3 Results and discussion

### 3.1 Characterization of the electroform

**Fig. 6:**
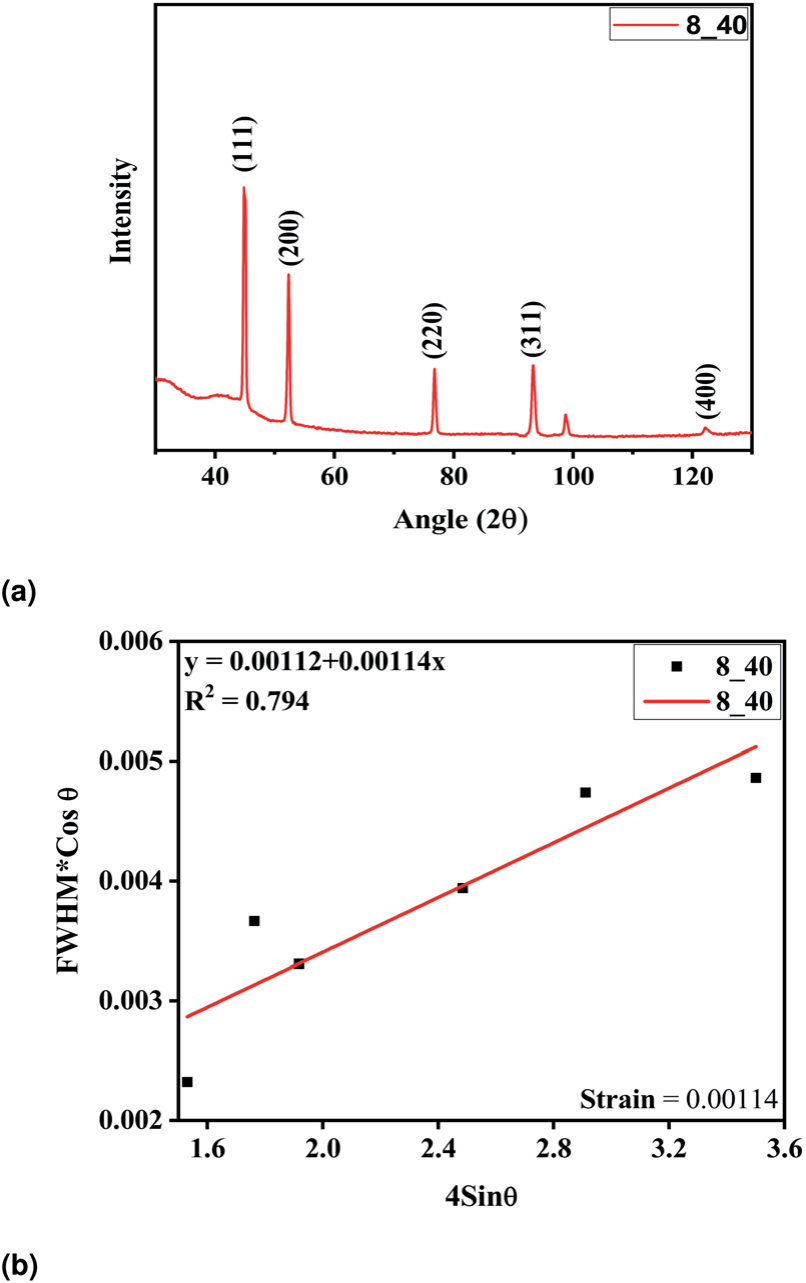
(a) XRD analysis of the electroformed Ni tubes, (b) W–H plot of the tube film.

The tubes were fabricated at optimum parameters of 40% of the duty cycle and 8 hours of deposition time. The electroformed nickel micro-tubes were characterized with the help of X-ray diffraction carried out with Cu Kα radiation (*λ* = 0.15405 nm). The tube source was operated at 40 V and 40 mA. The diffraction patterns were registered in the 2*θ* range of 30–110° and phase identification was done using JCPDS cards. The Ni tubes were deposited upon an aluminium mandrel and extracted by dipping in potassium hydroxide solution thereafter. But no extra diffraction peaks were detected, indicating that pure Ni was deposited. The intensities of the peaks and the narrow peak breadth as shown in Fig. 6a confirm that the deposition is of good quality with high crystallinity and a fine grain size. The high intensity of the (111) plane as compared to the other peaks indicate it to be the preferred orientation of the microcrystallites. The crystallite size was calculated from the well-known Scherrer formula^25^ and found to be 47.17 nm. The W–H plot in Fig. 6b demonstrates the minimum amount of strain induced in the films during the process of fabrication.

**Fig. 7:**
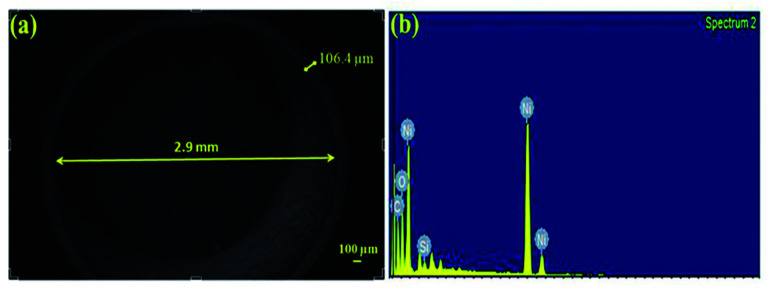
(a) Top view of the microtube, (b) EDAX analysis for the type of elements present in the microtube.

The tube films were characterized using FESEM to measure their structural integrity. The thicknesses of the tubes were found to be around 105 μm as shown in Fig. 7. The fabricated tubes with the above mentioned process parameters were found to possess an excellent hardness of 288 HV and a surface roughness of 1.92 μm. The hardness values were compared with other references in the literature for electrodeposited Ni. The hardness values of the samples were well above what has been cited in the literature which is around 200 HV.^26^ Hence the fabricated metallic tubes possessed good rigidity to be used further in the process of growth and development of the nanorods.

### 3.2 Characterization of the ZnO nanorods

**Fig. 8:**
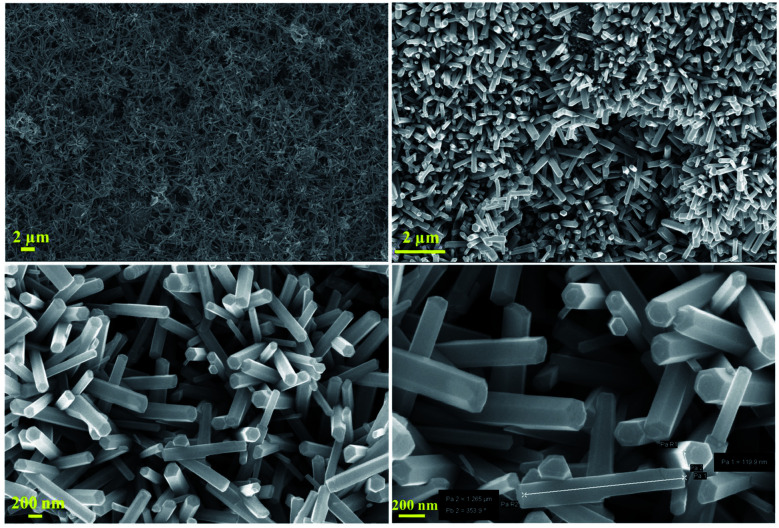
FESEM images of the developed nanorods.

**Fig. 9:**
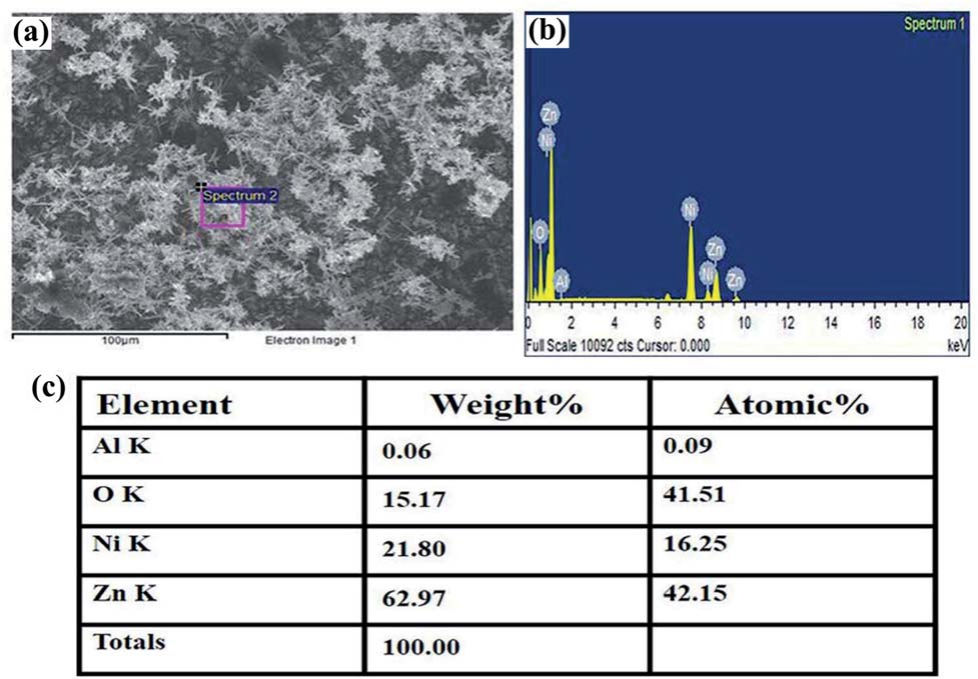
(a) The selected area for EDS analysis, (b) peaks showing the elements present, (c) the weight percentage of the elements.

The structural characterization of the fabricated nanorods on the Ni electroformed films was carried out using a field emission scanning electron microscope (ZEISS MERLIN compact) in conjunction with EDS. The FESEM images in Fig. 8 show the density and orientation of growth of the nanorods at various magnifications. The figures illustrate the vertical high-aspect ratio growth of the nanorods. Due to the surface morphology of the electroformed nickel films, the nanorods were arranged in flower configurations. Fig. 9a presents the EDS analysis of the ZnO nanorods grown on the Ni substrate with Fig. 9b and c showing the peaks and percentages of elements present respectively. The flower like morphology of the NRs was obtained thanks to the combination of HMTA and DI water. While the hydrolysis of HMTA provides a sufficient amount of ammonium ions, the use of DI water as the solvent causes complexation at higher rates thereby clustering the nanoseeds and resulting in uncontrolled growth to form long rods.

**Fig. 10:**
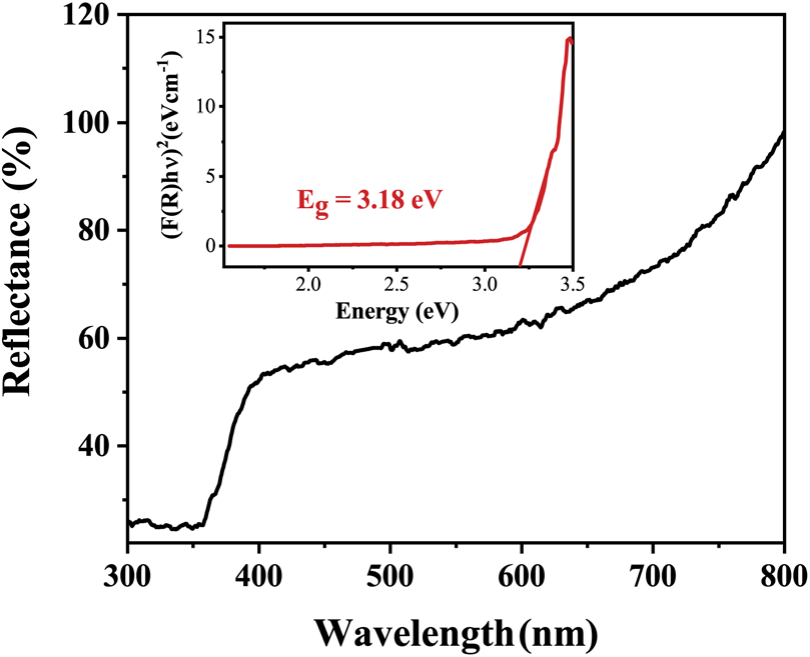
The UV-vis-DRS spectrum of ZnO nanorods plotted between reflectance (%) and wavelength. The inset shows the Tauc plot and band gap energy.

The band gap energy of the ZnO NRs was determined through the reflectance spectra obtained with respect to wavelength. The wavelength was varied in the range 300–800 nm. The UV-vis-DRS spectrum in Fig. 10 demonstrates a trailing edge to the spectrum in the near UV region followed by a sharp increase in reflectance typical of a band gap transition. The band gap energy can be determined from the reflectance spectra according to the theory proposed by Kubelka and Munk in 1931.^27^ The theory devised a transformation method of reflectance spectra to absorption spectra through the application of the Kubelka–Munk function (*F*(*R*)) which is given by the following equation.1
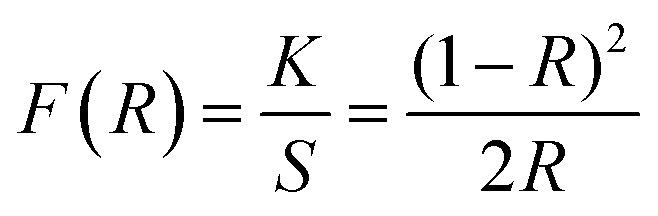
where *R* is the reflectance while *K* and *S* are the absorption and scattering coefficients respectively. *F*(*R*) can then be used in the place of α in the method derived by Tauc^28^ for the determination of the band gap using the absorption spectra. The resulting equation is given by:

2 (*F*(*R*)ℏ*ν*)^1/*γ*^ = B(ℏ*v* − *E*_g_)

where ℏ is the Planck constant, *ν* is the photon’s frequency, *E*_g_ is the band gap energy, and B is a constant. The value of *γ* depends on the nature of the semiconductor band gap transition and is 1/2 and 2 for direct and indirect band gap transitions respectively.

Fig. 10 shows the reflectance spectrum of ZnO (a direct band gap semiconductor) and the inset depicts the Tauc plot according to eqn (2). The figure demonstrates a steep increase in absorption with increasing photon energy. The *x*-axis linear interpolation of the Tauc plot gives a band gap energy of 3.18 eV for the synthesised ZnO nanorods.

### 3.3 Thermodynamic constraints of ZnO nanorod growth on the substrate

The growth of ZnO nanorods on any substrate depends on the substrate’s ability to facilitate the uniform distribution of the nanoseed layer. The nature of the substrate surface influences the deposition of the nanoseed layer which subsequently provides the nucleation sites for growth. The feasibility of the nanorod growth process depends on minimizing the Gibbs free energy. The Gibbs free energy change (Δ*G*) for nucleation is given as follows:

3 Δ*G* = 4/3π*r*^3^ (Δ*G*_v_) + 4π*r*^2^*γ*

where *r* is the radius of the embryo, *γ* is the interfacial energy, and Δ*G*_v_ is the change in Gibbs free energy due to the unit change in volume. The first term represents the gain in energy due to the creation of new volume while the second term is the loss in surface energy due to the surface tension of the new interface. The critical energy required for nucleation to occur can be derived by setting dΔ*G*/d*r* = 0 and is given as follows:

4 ΔG* = (16π*γ*^3^)/(3(Δ*G*_v_)^2^

**Fig. 11:**
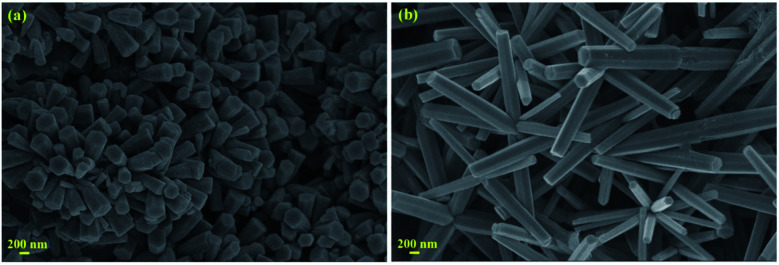
SEM images of the as-grown ZnO nanorods at different temperatures (a) 80 °C and (b) 90 °C.

The volumetric change in free energy (Δ*G*_v_) is represented as follows:5
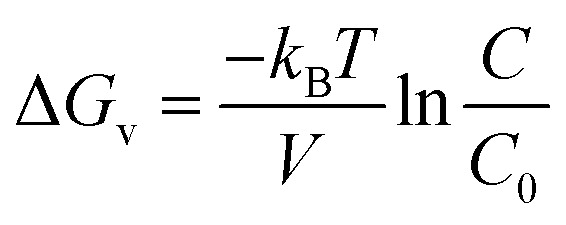
where *k*_B_ is the Boltzmann constant, *V* is the atomic volume, *T* is the temperature in kelvin, *C* is the zinc concentration at a specific time and *C*_0_ is the initial concentration of the zinc in solution. The nucleation and uniform growth of the nanorods is possible only when the Gibbs free energy is lower than or equal to the critical value (ΔG*). The coating of the nanoseed layer carried out in the first step of the process helps in lowering this thermodynamic barrier by providing enough nucleation sites. It was also found that prior annealing of the substrates at around 250–300 °C for the decomposition of zinc acetate was not essential. The growth of well aligned nanorods was obtained at around 90 °C. But nanorods grown at 80 °C had shorter lengths with larger diameters compared to the ones obtained at 90 °C as seen in Fig. 11. As a higher temperature results in the faster mobility and diffusion of ions, this leads to an increased length of the nanorods.

### 3.4 Photocatalytic study of the ZnO nanorods grown over tubes

**Fig. 12:**
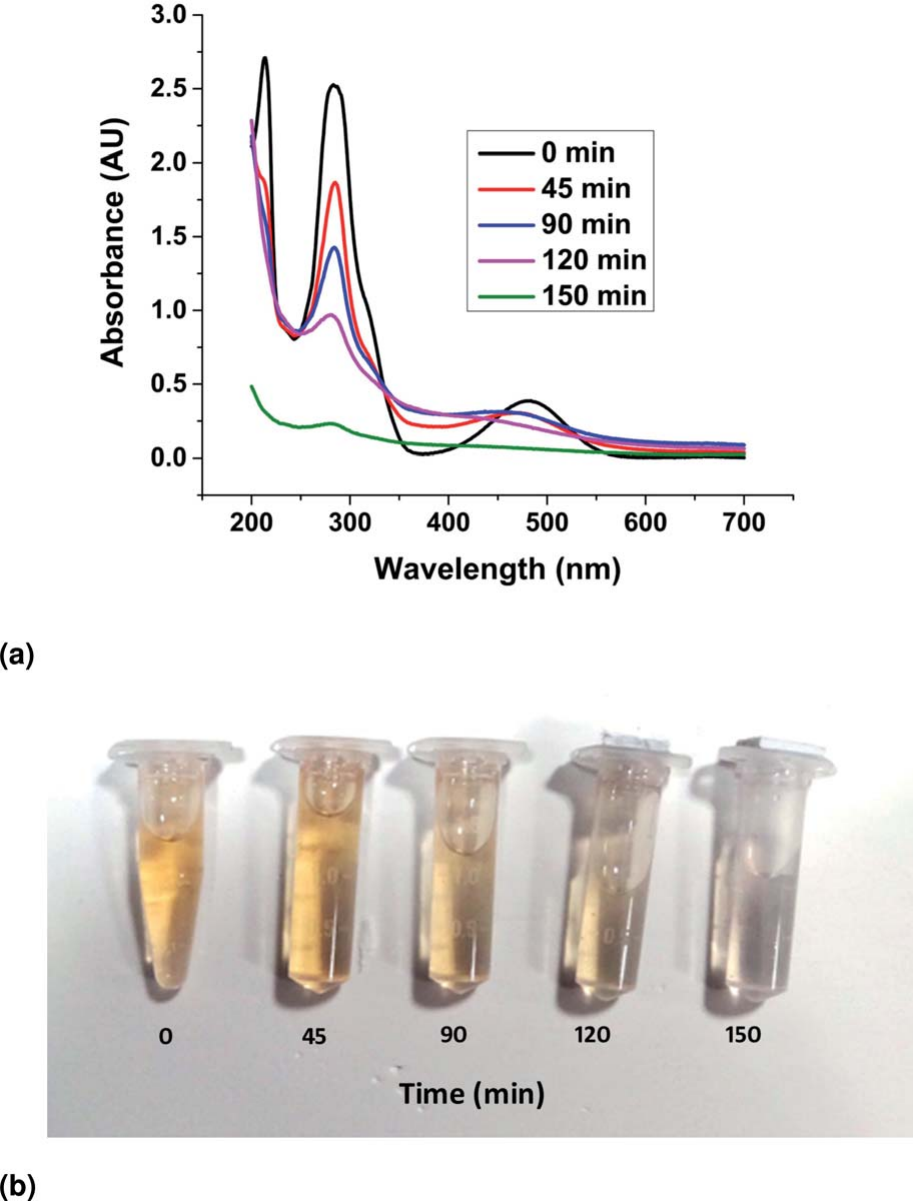
(a) UV–vis absorbance curves of the photocatalytic degradation. (b) The gradual de-coloration of the solution in the presence of the catalyst.

The UV–Visible absorption spectra of the EtBr aqueous solution irradiated with visible light in the presence of the ZnO nanorods were recorded and presented in Fig. 12a. The spectra show a characteristic maximum peak at 285 nm and another broad, low secondary peak ranging from 400 nm to 550 nm. Besides, an additional peak at 210 nm was detected, which also declined with increasing reaction time. The vertical decline in the absorbance peak represents the decreasing concentration of EtBr in the solution with an increase in irradiation time. This can be attributed to the photocatalytic degradation of EtBr in the solution due to the presence of the ZnO catalyst in the form of nanorods. The horizontal shift in the peaks can be explained by the presence of ZnO powder in the solution as the sample taken for UV–vis spectroscopy was not filtered to remove ZnO particles from the sample. It has also been observed that complete mineralization (initiated by the rupture of the phenanthridinum ring) of the EtBr solution happened in 150 min. The gradual change in colour of the EtBr solution and complete decolouration in 150 min have been demonstrated through the samples shown in Fig. 12b.

**Fig. 13:**
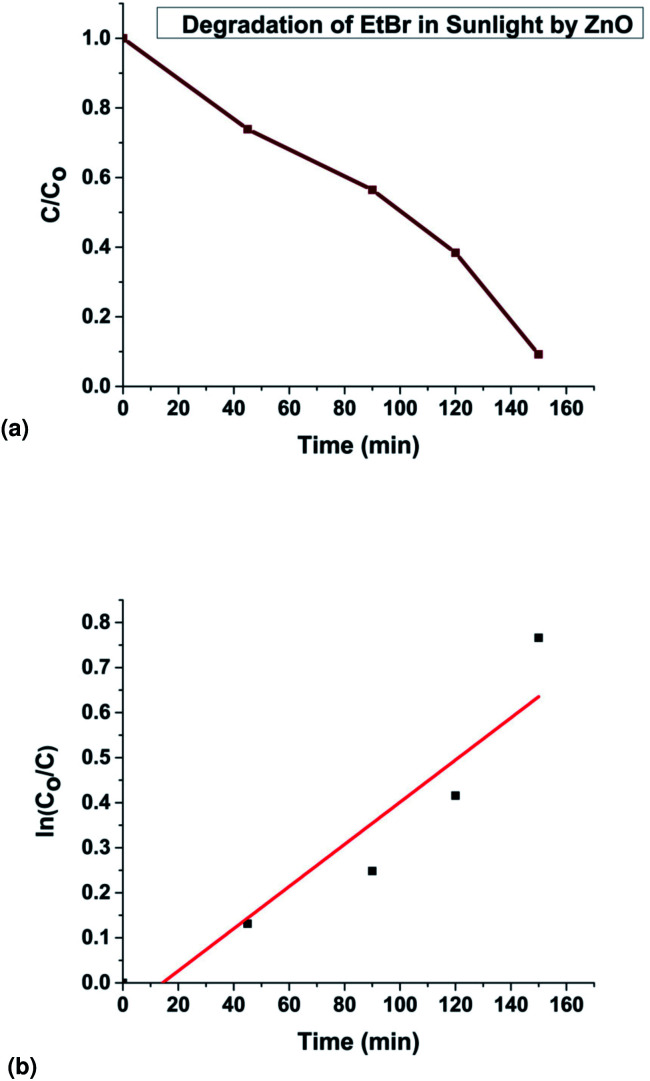
(a) Experimental data showing EtBr degradation in sunlight, (b) kinetics of the photodegradation of EtBr.

**Fig. 14:**
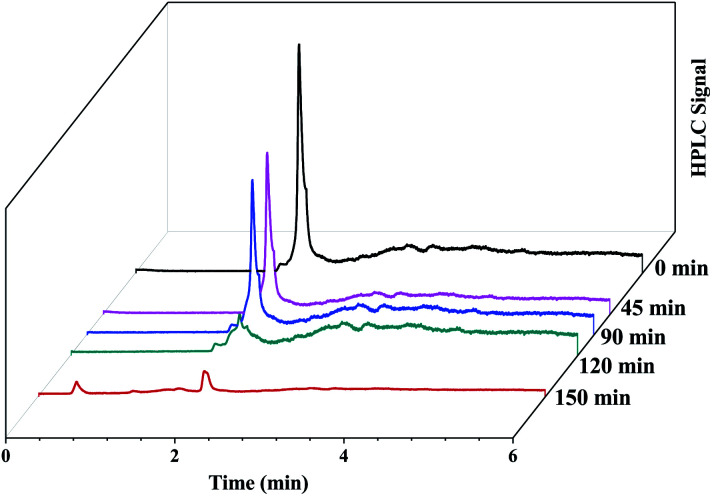
HPLC chromatograms of the EtBr dye solution irradiated in sunlight at different intervals of time in the presence of the photocatalytic substrate.

The fitting of the normalized maximum absorbance with respect to time indicates a decay as shown in Fig. 13a. The normalized concentration of the EtBr solution takes the place of the normalized absorbance according to the Beer–Lambert law which states that there is a linear relationship between the concentration and absorbance of the solution. Hence, *C*/*C*_0_ has been used instead of *A*/*A*_0_ where *C*_0_ is the initial concentration and *C* is the concentration at a particular time. Various studies have demonstrated that the photocatalytic degradation kinetics of several organic dyes illuminated on ZnO follows a Langmuir–Hinshelwood kinetics model.^29–31^ The first-order equation is given as below:6
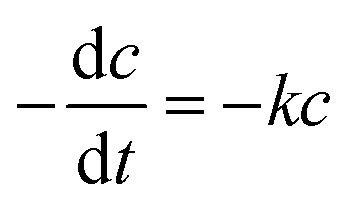
7
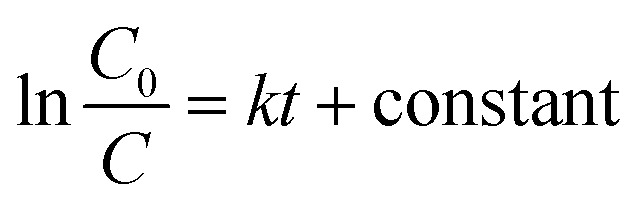
where *k* is the first-order rate constant that can be obtained from the graph of the linear regression kinetics line while *C*_0_ and *C* are the concentrations of the dye solution at time *t* = 0 and *t* respectively. Accordingly the degradation behaviour of EtBr is found to follow pseudo first-order kinetics as shown in Fig. 13b.The HPLC-UV detection of solutions at different irradiation times were evaluated and presented in Fig. 14. Calibration using a standard solution of EtBr at different concentrations was carried out and the chromatogram presents a single peak at the retention time of 2 min which corresponds to EtBr. As the irradiation time increases, the peak intensity at 2 min decreases indicating the degradation of EtBr in the presence of the developed substrate. 80% mineralization was achieved within 150 min of irradiation. A degradation efficiency of above 90% can be achieved at a higher intensity or with a longer irradiation time.

### 3.5 Removal of organic compounds

**Fig. 15:**
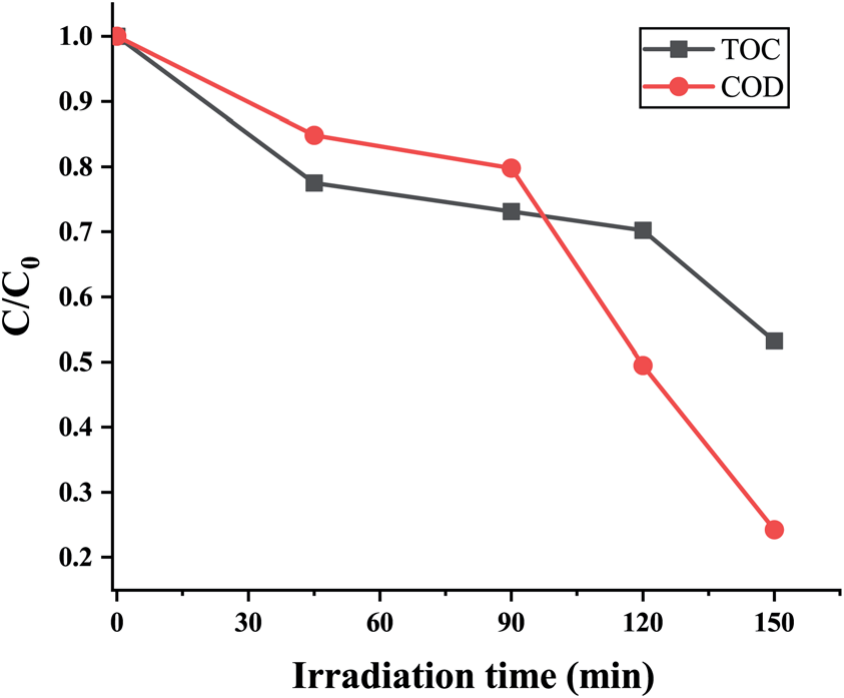
EtBr degradation process showing the decrease in TOC and COD at different irradiation times.

Chemical oxygen demand (COD) is the amount of oxygen consumed to oxidize organic pollutants in water which is a significant indicator of water quality. A decline in the value of COD demonstrates the degradation of organic compounds into simpler ones, thereby improving the quality of the water. While the COD value of 10 ppm EtBr solution was determined to be 79.2 mg L^−1^, the sample after being photodegraded for 150 min had a COD value of 19.2 mg L^−1^. The partial degradation may be because of some residual organic content in the sample due to a lowering of the sunlight intensity (100 mW cm^−2^). But a significant drop of 76% in the COD value can be attributed to photodegradation by the ZnO nanorods grown on the electroformed Ni tubular films. The mineralization efficiency *i.e.*, the conversion of organic compounds to inorganic species is signified by the TOC value. Fig. 15 indicates a maximum TOC decrease of 47%, signifying that 47% of the EtBr was oxidized to CO_2_ and H_2_O by the active species (·OH and ·O_2_^−^) at the end of the 150 minutes.

It can be noticed that the TOC decrease is somewhat lesser than the COD decrease. As the COD values can correspond to partial oxidation but incomplete mineralization of the organic molecules, it can be suggested that the organic carbon may have existed in a higher oxidation state after the photocatalytic treatment. The emergence of a peak at 210 nm in the UV–vis absorption spectra further indicates the formation of intermediate compounds. The attack of active radicals (·OH and ·O_2_^−^) on the phenanthridinium ring results in the de-colourization of EtBr with the formation of intermediate cyclic compounds. As previous works have suggested that de-colourization means the detoxification of the EtBr dye,^11,35^ it can be suggested that this work possesses great potential for the photocatalytic degradation of EtBr through a green and economically inexpensive route.

The efficiency of the developed system is evident from the fact that complete de-colourization of the EtBr dye happened within 150 minutes. Table 1 compares existing photodegradation systems using various catalysts with the present research. The developed system with a comparatively lower amount of the ZnO catalyst could achieve complete decolourization without any metal doping. The research work carried out by Han *et al.*^34^ involved the synthesis of a complex nano-composite, though it demonstrated a promising result of 60 minutes degradation time. Also the present research showcases a 47% TOC removal efficiency as compared to 45% for Han *et al*’s .

**Table 1:** Comparison between existing and present research for EtBr degradation

Catalyst used	Amount of catalyst (gL^−1^)	EtBr concentration (ppm)	Irradiation time (mins)	References
TiO_2_	1	80	195	M. Faisal *et al.*^32^
4% Zr doped TiO_2_	—	10	120	S. Swetha *et al.*^33^
C, Fe doped TiO_2_	1	20	120	Lavand *et al.*^13^
Attapulgite-@Fe_3_O_4_ with H_2_O_2_	1.5	80	60	Han *et al.*^34^
ZnO on Ni tube	0.2	10	150	Present research

### 3.6 Computational studies on the adsorption of EtBr on ZnO NRs

**Fig. 16:**
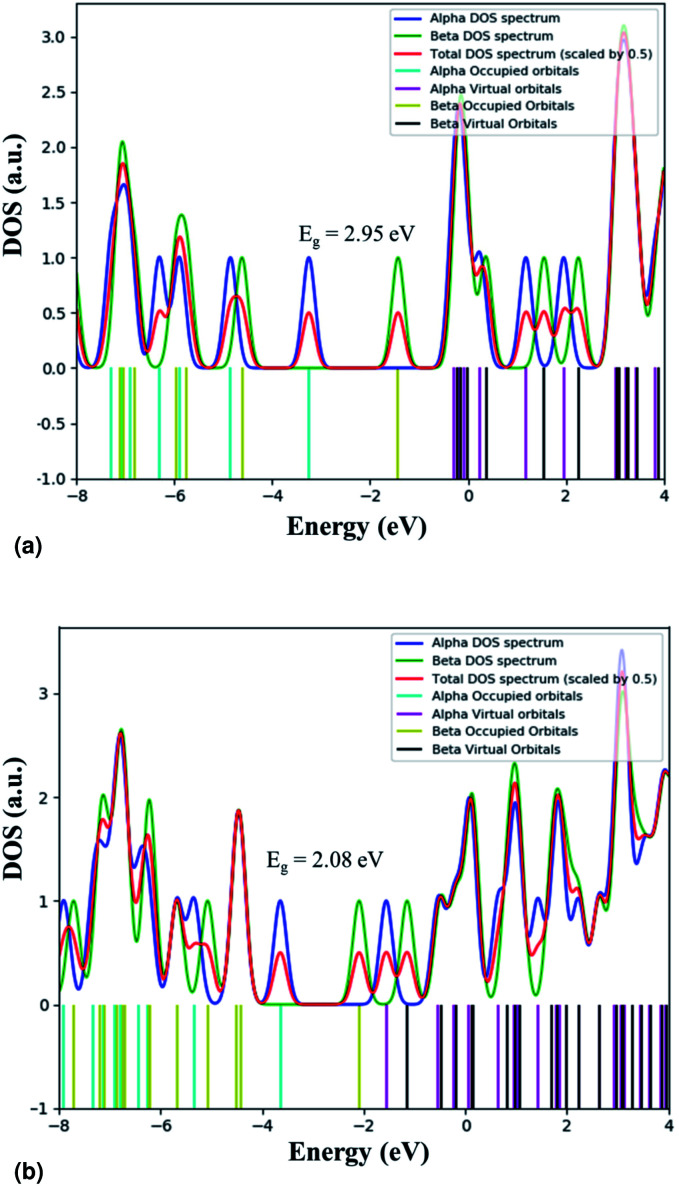
Total DOS plots for (a) EtBr and (b) ZnO–EtBr.

We calculated the adsorption energy of EtBr on the ZnO NR unit cell and found that *E*_ads_ is −645.7 kJ mol^−1^. The calculated value of *E*_ads_ is in the range of the chemical adsorption energies. This means that EtBr is strongly adsorbed on the ZnO NR surface. This strong adsorption causes the negative charge transfer from EtBr to ZnO. Table 2 summarizes the calculated HOMO and LUMO charges of the EtBr before and after the adsorption. The energy values of HOMO and LUMO and the difference between the energies of HOMO and LUMO are used to evaluate the chemical activity of EtBr adsorption on ZnO NRs. If the value of the difference between the energies of HOMO and LUMO is low, it implies that the chemical activity is high for that atomic condition and *vice versa*.^36^ It can be seen that the difference between the energies of HOMO and LUMO is lower for ZnO–EtBr in comparison to EtBr. Hence, the adsorption is favourable in this case. The electronic modulations were also scrutinized by exploring their band gap derived from their density of state (DOS) plots (shown in Fig. 16). The band gap of EtBr computed here comes out as 2.95 eV. The decrement in the band gap to 2.08 eV was noticed after the adsorption of EtBr on the ZnO NRs. The lowering of the band gap is attributed to the appearance of new energy levels that represent the mixing of the electronic states of the dye molecules and ZnO NRs.

**Table 2:** HOMO and LUMO energies of EtBr before and after adsorption.

		*E*_HOMO_	*E*_LUMO_	*E*_HOMO_–*E*_LUMO_
EtBr	α	−3.25 eV	−0.30 eV	−2.95 eV
	β	−4.62 eV	−1.43 eV	−3.19 eV
ZnO–EtBr	α	−3.64 eV	−1.56 eV	−2.08 eV
	β	−4.42 eV	−2.09 eV	−2.33 eV

## 4 The proposed degradation mechanism

The fact that the efficiency of ZnO as a catalyst for the photocatalytic degradation of organic dyes is superior compared to other semiconductor catalysts, has been corroborated by other research.^37^ The quantum efficiency of ZnO is significantly higher than that of other semiconductors because of its large band gap of 3.17 eV. But the broad band gap of ZnO (3.17 eV) makes it efficient for absorbing UV light (*λ* < 400 nm) and UV light accounts for only 5–6% of sunlight while the percentage of visible light is around 50% which is substantially higher. Hence, to exploit visible light as a source for degradation, efforts have been directed towards developing ZnO-based visible-light-active photocatalysts. While one approach is by bulk doping the semiconductors, thereby reducing the band gap, the present approach has aimed for the development of nanostructures with a high surface area and the involvement of a coloured dye.

In this approach the semiconductor is not used directly to initiate the degradation process but as a medium to transfer the electron from the excited dye to the electron acceptor (*e.g.* O_2_). As most of the dyes maintain equilibrium in a homogeneous solution irradiated by visible light, the photoinduced charge separation is rather unlikely. But the higher surface area of the semiconductor nanostructures not only provides adsorption sites for the dye but also facilitates efficient electron injection into the conduction band. The wide and continuous energy distribution in the conduction band of a semiconductor renders enough unoccupied electron-acceptor sites. These trap sites possess less energy than the conduction band edge and provide ample space for the adsorption of reaction substrates such as O_2_ or organic compounds. Hence, the organic coloured dye is the one which gets excited by the incidence of visible light.^38^ The present system consists of a high surface area photocatalyst in the form of nanorods (NR) along with a coloured dye which is adsorbed on the NR surface. The excited dye species adsorbed on the surface of the semiconductor oxide can then inject electrons into the conduction band thus generating conduction band electrons as presented in the following equations. The explained mechanism has been validated by DFT calculations showing the superior adsorption of the EtBr dye on the ZnO photocatalyst surface. The mixing of the electronic states of the EtBr dye and ZnO NRs was also noticed through the lowering of the band gap energy which can be attributed to the formation of new energy levels.EtBr + ℏ*ν* → EtBr*EtBr* + ZnO → EtBr^+^ + ZnO(e^−^)

**Fig. 17:**
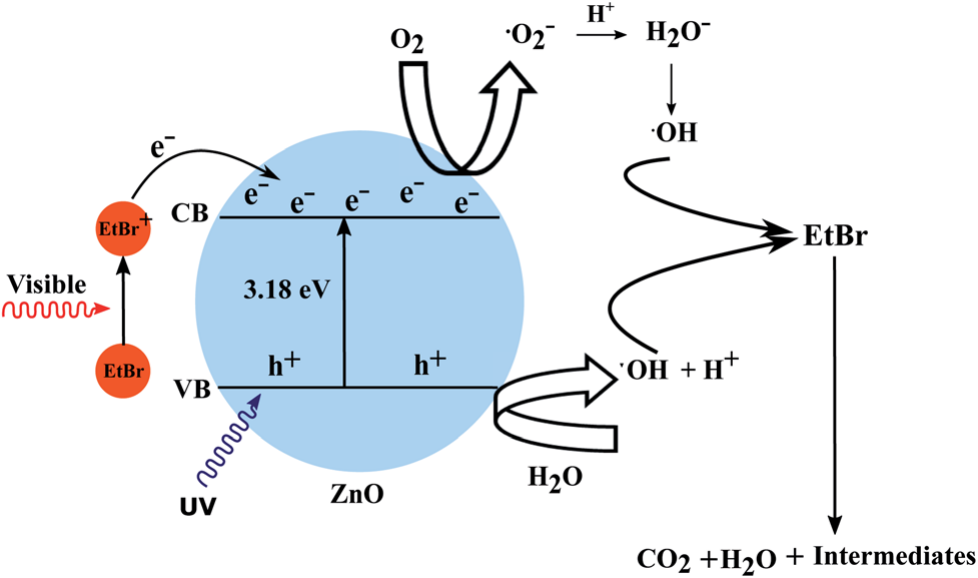
The proposed mechanism to explain the photocatalytic degradation of EtBr.

The superoxide radical anions (·O_2_^−^) and the hydroxyl radicals (·OH) play a dominant role in the redox degradation of the organic compound. Hence the direct oxidation of organic pollutants through the photogenerated holes has a limited possibility of occurring, though it can not be completely ignored. The direct excitation of the semiconductor may have happened to some extent because of the UV component of sunlight in the present work. This process is useful for the mineralization of coloured organic compounds with the utilization of visible light as a source of energy. Colourless organic pollutants can also be degraded by using other coloured species as antennae. The explained mechanism has been depicted in Fig. 17.

## 5 Conclusions

This work demonstrates the successful growth and development of ZnO nanorods on nickel tubular thin films. The metallic substrates have been obtained through using in-house developed electroforming equipment for the fabrication of thin hollow symmetrical structures. The tubular substrates were responsible for providing a high surface area-to-volume ratio for the good distribution of ZnO nanorod growth. The FESEM studies of the ZnO nanostructures confirmed their dense growth and showed the hexagonal nanorods to have a diameter of 100 nm. The as-grown ZnO nanorods on the electroformed nickel tubular films have been found to possess enhanced photocatalytic performance for the degradation of EtBr dye. The complete mineralization of the dye within 150 min shows the efficacy of the catalyst to be used for waste water treatment in processes like electrophoresis. A COD removal efficiency of 76% and the reduced peak of EtBr in the HPLC chromatogram confirms its degradation in the presence of the as-grown nanorods. To understand the adsorption properties of the EtBr dye on ZnO NRs, DFT calculations were performed. The DFT results show a strong adsorption of EtBr due to the negative charge transfer from EtBr to ZnO which further explains the proposed photodegradation mechanism.

## Author contributions

HJB performed all experimental work, AY performed the theoretical studies, AG formulated the research problem, HJB, AG and AY analyzed the results, and HJB, AY, PRP and AG wrote the manuscript.

## Conflicts of interest

There is no conflict of interest among the contributing authors.

## Acknowledgements

The authors would like to acknowledge the Centre for Advanced Scientific Equipment (CASE) facility at IIT Jodhpur and the Environmental Engineering Laboratory at IIT Bhubaneswar for providing characterization facilities. Furthermore, the authors also acknowledge the help from IMMT, Bhubaneswar with the characterization.

## Supplementary Material
